# Occurrence of Multidrug Resistant Extended Spectrum Beta-Lactamase-Producing Bacteria on Iceberg Lettuce Retailed for Human Consumption

**DOI:** 10.1155/2015/547547

**Published:** 2015-05-03

**Authors:** Natasha Bhutani, Chithra Muraleedharan, Deepa Talreja, Sonia Walia Rana, Sandeep Walia, Ashok Kumar, Satish K. Walia

**Affiliations:** ^1^Department of Biological Sciences, Oakland University, 375 Dodge Hall of Engineering, Rochester, MI 48309, USA; ^2^Department of Ophthalmology, Kresge Eye Institute, Wayne State University School of Medicine, 4717 Saint Antoine Street, Detroit, MI 48201, USA; ^3^Department of Gastroenterology, Henry Ford Health System, Detroit, MI 48208, USA

## Abstract

Antibiotic resistance in bacteria is a global problem exacerbated by the dissemination of resistant bacteria via uncooked food, such as green leafy vegetables. New strains of bacteria are emerging on a daily basis with novel expanded antibiotic resistance profiles. In this pilot study, we examined the occurrence of antibiotic resistant bacteria against five classes of antibiotics on iceberg lettuce retailed in local convenience stores in Rochester, Michigan. In this study, 138 morphologically distinct bacterial colonies from 9 iceberg lettuce samples were randomly picked and tested for antibiotic resistance. Among these isolates, the vast majority (86%) demonstrated resistance to cefotaxime, and among the resistant bacteria, the majority showed multiple drug resistance, particularly against cefotaxime, chloramphenicol, and tetracycline. Three bacterial isolates (2.17%) out of 138 were extended spectrum beta-lactamase (ESBL) producers. Two ESBL producers (T1 and T5) were identified as* Klebsiella pneumoniae*, an opportunistic pathogen with transferable sulfhydryl variable- (SHV-) and TEM-type ESBLs, respectively. The DNA sequence analysis of the *bla*_SHV_ detected in* K. pneumoniae* isolate T1 revealed 99% relatedness to *bla*_SHV_ genes found in clinical isolates. This implies that iceberg lettuce is a potential reservoir of newly emerging and evolving antibiotic resistant bacteria and its consumption poses serious threat to human health.

## 1. Introduction

Antibiotic use in the agriculture, aquaculture, and livestock industries has led to the emergence of antibiotic resistance genes (ARG) in the environment [1]. The use of antibiotics as growth promoters in the agriculture industry is particularly egregious; this use accounts for nearly half the 50 million pounds of antibiotics produced in the United States each year [2]. In addition, most antibiotics consumed by livestock or poultry are excreted as biologically active metabolites which can then select for and promote the growth of antibiotic resistant bacteria (ARB) [1]. Both antibiotics and ARB have been detected in animal waste, aquaculture, wastewater, river sediments, and farmland soil [3–6]. Low levels of antibiotics have been detected in the leaves of growing plants cultivated using antibiotic contaminated soil, water, and sediments, further selecting and promoting the growth of ARB on green leafy vegetables [7]. The concurrence of the selection of ARB on green leafy vegetables and the increasing occurrence of foodborne pathogens on fresh produce is worrying.

There have been several recent outbreaks of foodborne illnesses associated with the consumption of fresh green leafy vegetables [8]. In 2011, 3842 human infections in Germany with enteroaggregative hemolytic* E. coli* O104:H4 causing hemolytic uremic syndrome were associated with fenugreek seeds [9]. In the United States, spinach grown in Monterey County, California, infected with* E. coli* O157:H7 caused 15 deaths and over 100 hospitalizations [10]. Similarly, there was an outbreak of Shiga-toxin-producing* E. coli* O157 on lettuce in Netherlands and Iceland in 2007, which resulted in at least 50 illnesses [11], and an outbreak of* Shigella sonnei* associated with iceberg lettuce in Europe in 1995 which resulted in over 100 confirmed cases of shigellosis [12]. Of particular interest to our study is the outbreak of* E. coli* O145 HUS associated with shredded romaine lettuce purchased in Michigan and Ohio in 2010 [13]. The increasing prevalence of foodborne pathogens on green leafy vegetables has been previously been attributed to the bacterial ability to be internalized from contaminated manure or water into the leafy plant tissue [14], specifically within the stomata [15]. These foodborne infections often cause gastrointestinal illnesses and severe cases are generally treated using beta-lactam antibiotics [16], particularly third and fourth generation cephalosporins. However, this treatment option is much less effective if the foodborne pathogens are ARB and extended spectrum beta-lactamase (ESBL) producers [17], as ESBL enzymes hydrolyze this newest generation of beta-lactam antibiotics.

In recent years, a growing number of studies have shown the emergence of bacterial strains resistant to beta-lactams and the main underlying mechanism is the production of beta-lactamase enzymes. The increasing prevalence of ESBL producers in particular is troubling, given their high correlation with multidrug resistance (defined as resistance to three or more classes of antibiotics) [18]. Additionally, the genes encoding beta-lactamases and other antibiotic resistance genes are often found on mobile genetic elements such as transposons and plasmids [19–21]. Thus, the ARB genes can be easily transferred from saprophytic bacteria to opportunistic pathogens. This transfer has already been demonstrated for ampicillin resistance encoded by ESBL genes in environmental bacteria found on lettuce [22] and spinach [23].

Since iceberg lettuce (IBL) is usually consumed rawly and has been identified as a source of foodborne pathogens by the World Health Organization, this study examined whether IBL leaves harbor ARB, specifically ESBL-positive bacteria. Bacteria found on green leafy vegetables are generally assumed to be harmless, but the expression of antibiotic resistance may suggest that the bacteria have other virulence properties [24]. Therefore, potential pathogens, like ESBL-producing* K. pneumoniae*, were further characterized for their ability to cause inflammation and cytotoxicity on human intestinal epithelial cell line CACO-2 [25]. Here we demonstrate that IBL retailed in Michigan carry ESBL-producing multidrug resistant bacteria with resistance genes on self-transmissible plasmids.

Some of these results were presented at the 52nd Interscience Conference on Antimicrobial Agents and Chemotherapy, San Francisco, California, Poster Session 088, Presentation C2-703.

## 2. Materials and Methods

### 2.1. Bacteriological Analysis of Iceberg Lettuce

In this pilot study, nine iceberg lettuce samples were purchased from local retail markets in Rochester, MI area. The lettuce samples were purchased from national distributors and all samples originated from the Salinas valley in California, a major leafy vegetable-producing area in the United States [26]. The lettuce samples were stored at 4°C and processed for bacteriological analysis within 24 h of purchase. The samples were processed by first removing the outer leaves and then weighing 25 g of each sample and placing it in a sterile stomacher bag with 100 mL of 0.1% peptone water. The stomacher bag was sealed and kneaded in a stomacher at 150 rpm for 20 min. The resulting wash was then serially diluted 4 logs in 0.1% peptone water and 0.1 mL of log dilution was plated on tryptic soy agar (TSA, Becton Dickenson) and MacConkey (MAC, Becton Dickenson) plates with and without antibiotics [27]. The antibiotics used included CTX 64 *μ*g/mL, CIP 4 *μ*g/mL, GEN 16 *μ*g/mL, and TET 16 *μ*g/mL [28]. Antibiotic powders were purchased from Becton Dickenson. The quality of the antibiotic plates was confirmed using the quality control strains* E. coli* ATCC 25922 and* Pseudomonas aeruginosa* ATCC 27853. The number of CFU per gram of iceberg lettuce was determined for each IBL sample in order to determine the microbiological quality of the IBL samples. Single isolated bacterial colonies with distinct colony morphology and pigment production were randomly selected, picked, purified, and stored at −80°C for further analysis.

### 2.2. Biochemical Identification and Antibiotic Susceptibility Testing

The bacterial isolates from iceberg lettuce were identified using the BD Phoenix Automated Microbiology system [29] which showed correlation with manual identification methods [30]. Briefly, the Phoenix ID broth was inoculated with isolated colonies of bacteria and the bacterial suspension was adjusted to 0.5 optical density at 540 nm with Phoenix AST broth using Phoenix Auto Processor. The Phoenix NMIC/ID-124 combo panels were loaded onto the Phoenix 100 system and samples were processed according to the manufacturer's instructions. Each bacterial isolate was also tested against a panel of six antibiotics using Kirby-Bauer disk diffusion method following CLSI guidelines [28]. The antibiotic disks (Becton Dickenson) used were CTX 30 *μ*g, cefotaxime/clavulanic acid (CTX-CLA) 30/10 *μ*g, TET 30 *μ*g, chloramphenicol (CHL) 30 *μ*g, GEN 10 *μ*g, and CIP 5 *μ*g. Possible ESBL-positive bacteria were identified by their keyhole formation between the CTX and CTX-CA disks and confirmed using the CLSI phenotypic confirmatory test with ceftazidime (CAZ) 30 *μ*g, ceftazidime/clavulanic acid 30/10 *μ*g (CAZ-CLA), CTX, and CTX-CLA disks [31].* K. pneumoniae* ATCC 700603 and* E. coli* ATCC 25922 were used as positive and negative controls, respectively.

### 2.3. Genomic DNA Extraction and PCR Amplification

Total genomic DNA was extracted from each suspected ESBL-positive strain using a boiling method [32]. For the amplification of the gene sequences, each 50 *μ*L PCR reaction mixture contained 2 *μ*L of extracted DNA, 1 picomol of each primer, and 1X Taq-Pro Red Complete master mix (Denville Scientific, Metuchen, NJ) containing a final concentration of 1.5 mM MgCl_2_. The primer sequences, annealing temperature, and expected size of the amplicon for ESBL gene sequences are summarized in Table 1. The PCR products were separated by electrophoresis on a 0.7% agarose-TAE gel containing ethidium bromide and photographed using UV light.* K. pneumoniae* ATCC 700603 and* E. coli* ATCC 25922 were used as positive and negative controls, respectively, for SHV [33].* K. pneumoniae* ATCC 700603 served as the positive control for the 16S rRNA amplification. PCR products that resulted in a band matching the expected size were purified using the QIAGEN PCR Products Purification kit following the manufacturer's instructions. The purified products were then cloned using the pGEM-T Easy Vector I system (Promega, Madison, WI) and sequenced in the ABI Prism 3730 DNA Analyzer (Applied Biosystems) at Wayne State University. The resulting sequences were aligned and compared to known beta-lactamase gene sequences in the BLAST databases at the NCBI website. A neighbor-joining phylogenetic tree was constructed in BioEdit using the bootstrap analysis run by the ClustralW Multiple Alignment tool.

### 2.4. Conjugal Transfer of Antibiotic Resistance to* E. coli*

Conjugal transfer experiments were done to determine whether beta-lactam resistance can be transferred from the donor environmental isolate to the sodium azide resistant recipient* E. coli* J53. Log cultures of donor and recipient were mixed in 1 : 1 ratio in 4 mL fresh TSB and the mixture was incubated at 25°C and 37°C for 24 h without shaking. The conjugal mix (0.1 mL) was spread on eosin methylene blue (EMB) agar containing 100 *μ*g/mL sodium azide and 0.25 *μ*g/mL ciprofloxacin. The transconjugants grown on selective media were purified and further tested for cotransference of antibiotic resistance and ESBL using the phenotypic disk diffusion tests recommended by CLSI [28].

### 2.5. Molecular Analysis, Isolation, and Hybridization Analysis of Plasmids

The plasmid DNA was extracted from* K. pneumoniae* ATCC 700603,* K. pneumoniae* T1,* K. pneumoniae* T5,* S. marcescens* M5, azide resistant* E. coli* J53,* E. coli* V517, and the transconjugants* E. coli* KC1, TC1, and TC5 using the QIAGEN Midiprep kit. The manufacturer's directions were followed for the purification of large, low copy number plasmids. The DNA was separated on a 1.0% agarose-TAE gel electrophoresed at 70 V for 1.5 h and then transferred to a positively charged nylon membrane (Roche) using the manufacturer's directions. Hybridization was carried out at 54°C using an 865 bp digoxigen-labeled *bla*_SHV_ probe created using the PCR DIG-labeling kit (Roche) and the ESBL gene primers listed in Table 1 following the manufacturer's instructions.

### 2.6. Pulsed Field Gel Electrophoresis of Environmental and Clinical Strains of* K. pneumoniae*

Pulsed field gel electrophoresis was performed on T1 and T5 to determine the clonal relationship between these two environmental* K. pneumoniae* strains and clinical* K. pneumoniae* strains. The clinical strains used were isolated from a variety of sources in 2011, including urine, sputum, and wounds. The genomic DNA was prepared using standard procedures and then digested using XbaI (New England BioLabs) [36]. The resulting restriction patterns were interpreted using the criteria proposed by Tenover et al. [37] and our recent study [31].

### 2.7. Statistical Analysis

Student's *t*-tests were used to determine if there were any significant differences in the total bacterial counts and the ARB count for the iceberg lettuce samples and if there were significant differences in cytokine production between clinical and environmental strains. A one-way analysis of variance (ANOVA) followed by Tukey's post hoc test was performed to evaluate the differences in resistance patterns of the bacterial isolates. Statistical tests were performed using Minitab 16 and Microsoft Office Excel.

### 2.8. Nucleotide Sequence Accession Numbers

The nucleotide sequence of *bla*_SHV_ reported in this study has been deposited in GenBank under the accession number JX045654.

## 3. Results

### 3.1. Bacteriological Analysis of Iceberg Lettuce

In this preliminary study 9 IBL samples were purchased from the local Rochester retail market between November 2011 and February 2012. The total bacterial plate count was determined for each IBL sample and the average viable plate count was 2.74 × 10^6^ ± 3.29 × 10^6^ (mean ± standard deviation, CFU/g). The average bacterial count for the total CTX-resistant community was 1.54 × 10^5^ ± 2.79 × 10^5^ (mean ± standard deviation, CFU/g), while the average bacterial count for the total tetracycline-resistant community was 2.89 × 10^2^ ± 5.75 × 10^2^ (mean ± standard deviation, CFU/g). No bacteria grew on the plates containing high concentrations of GEN and CIP. All of the plates containing antibiotics had significantly lower plate counts (*P* value < 0.05) than those without antibiotics. Given that the microbiological safety standard for green leafy vegetables is 10^8^ CFU/g, this suggests that all of the IBL samples were microbiologically safe for consumption. The predominant microbial community on iceberg lettuce was determined by cultivation of bacteria on TSA and MAC plates.

### 3.2. Biochemical Identification and Antibiotic Susceptibility Testing

The BD Phoenix Automated Microbiology system uses biochemical tests to identify bacteria and a total of 50 cultivable bacterial isolates were identified. The BD Phoenix identification system showed the presence of* Achromobacter* species,* K. pneumoniae*,* Mannheimia haemolytica*,* Pantoea agglomerans*,* Pseudomonas fluorescens*,* Pseudomonas putida*,* Pseudomonas oryzihabitans*, and* S. marcescens*.* Pseudomonas* species was the most predominant bacteria found on IBL, as seen in Table 2. All nine IBL samples contained drug resistant bacterial isolates. Among the 138 bacterial isolates tested, 86% were resistant to CTX, 83% to CHL, 44% to TET, 5% to CIP, and 4% to GEN. In addition, the isolates showed different antibiotic resistant patterns. 6.5% were resistant to only one antibiotic; 40.6% were resistant to 2 antibiotics (CTX, CHL); 40.6% were resistant to 3 antibiotics (CTX, TET, and CHL); 0.71% was resistant to 4 antibiotics; and 0% was resistant to 5 antibiotics (Table 3). Three of the nine strains resistant to only one antibiotic were resistant to CTX. Only 3 (2.1%) were detected as ESBL producers. It is interesting to note that all 3 ESBL-positive strains were isolated from a single IBL sample. A one-way ANOVA on the resistance patterns of these isolates yielded highly significant variation among the iceberg lettuce samples (*P* value < 0.001). The Tukey test for the separation of means demonstrated that the* K. pneumoniae* isolates T1 and T5 had significantly higher antibiotic resistance than the other bacterial isolates. Both of these isolates had the antibiotic resistance pattern CTX CHL GEN CIP, and the third ESBL-producing isolate,* S. marcescens*, demonstrated resistance against CTX and CIP.

### 3.3. Sequencing and Phylogenetic Analysis of ESBL Genes

**Figure 1:**
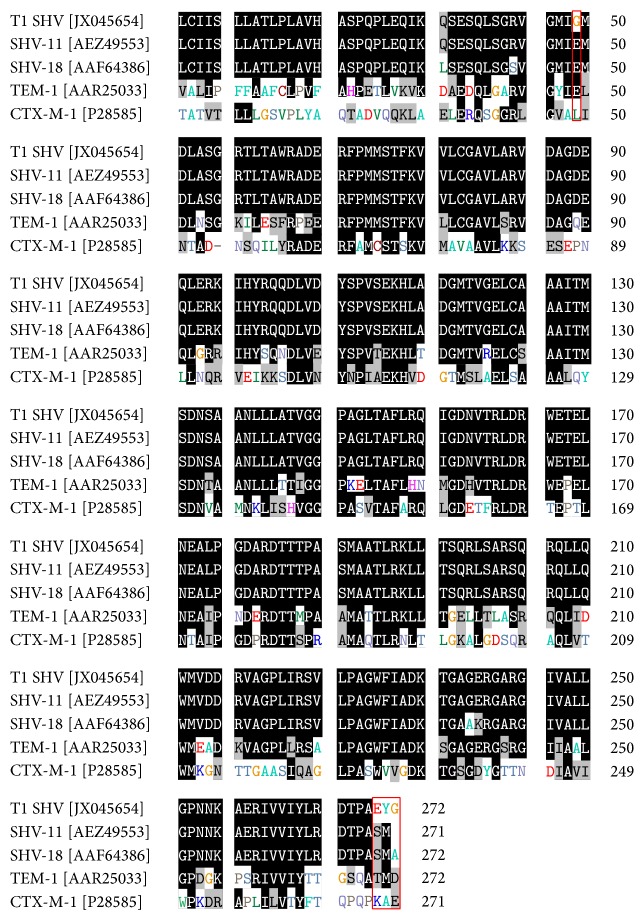
Amino acid sequence analysis of T1 SHV and other ESBLs. Amino acid substitutions are highlighted in red boxes. GenBank accession numbers are given in brackets and residue numbers are given in terms of the previously characterized SHV-11 from a clinical isolate in Austria (GenBank accession number AEZ49553).

**Figure 2:**
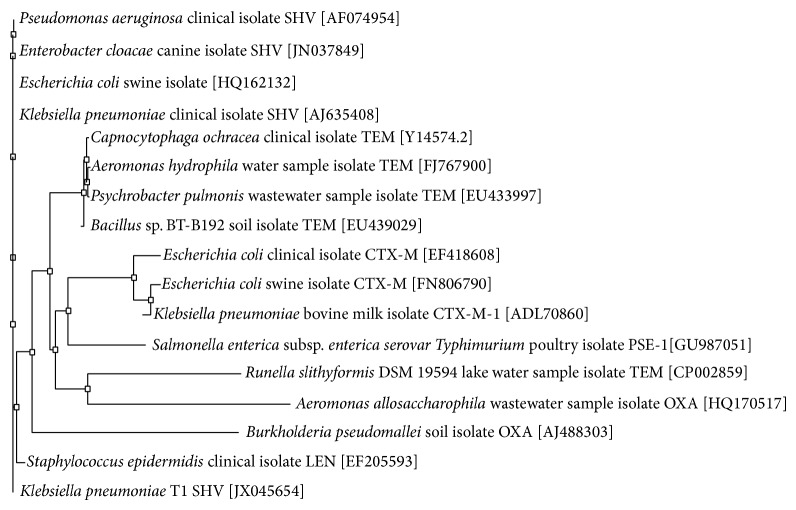
Phylogenetic tree of ESBL sequences. The phylogenetic tree represents a majority rule consensus tree based on protein similarity using neighbor joining. Bootstrap values (total 100) are calculated with neighbor joining and maximum likelihood methods.* Klebsiella pneumoniae* SHV is the outgroup.

The three ESBL-positive strains were designated T1, T5, and M5. T1 and T5 were both* K. pneumonia* isolates which contained SHV-type and TEM-type ESBLs, respectively. M5 was a* S. marcescens* isolate that contained a CTX-M-1 beta-lactamase, as detected by PCR. The ESBL sequences amplified from these three isolates were compared against known sequences using the NCBI database BLASTn (http://www.ncbi.nlm.nih.gov/blast/Blast.cgi#). A unique 751 bp sequence from one* K. pneumoniae* (T1) strain was 99% identical with positions 44–799 of an 852 bp SHV-11 sequence from a clinical isolate of* K. pneumoniae* from Austria (GenBank sequence accession number JN676837) (Figure 1). This sequence is identical to the originally reported sequence except for a single nucleotide change (GAA→GGA at position 131) which encoded for a change in amino acid from glutamic acid to glycine at position 44 and a single nucleotide insertion (CGA→CCGA at position 792) which encoded for a change in 3 amino acids (S→E at position 265, M→Y at position 266, and A→G at position 267). Unlike the ESBL gene sequence from T1, the ESBL gene sequences from T5 and M5 were identical to previously characterized ESBLs. The phylogenetic analysis of SHV-11 amino acid sequences derived from ESBL genes present on pOU11 revealed the divergence from the existing sequences indicating an emergence of a variant of SHV gene family (Figure 2).

### 3.4. Conjugal Transfer of Antibiotic Resistance to* E. coli*

**Figure 3:**
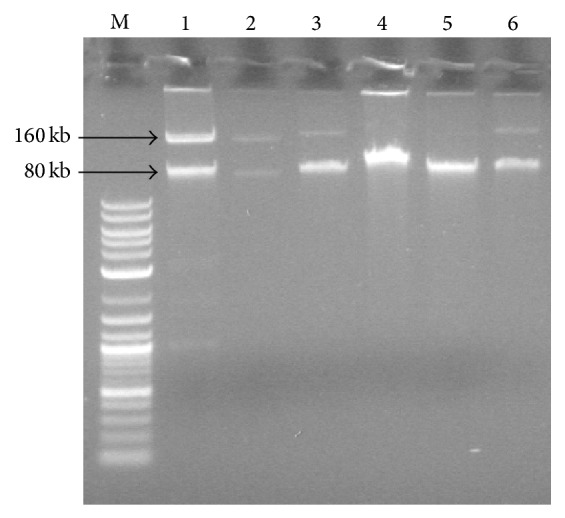
Plasmid profile of donors and transconjugants. Lane M: 1 kb linear DNA marker (New England BioLabs), Lane 1:* K. pneumoniae* ATCC 700603, Lane 2:* E. coli* transconjugant of* K. pneumoniae* ATCC 700603 mated at 37°C, Lane 3:* E. coli* transconjugant of* K. pneumoniae* ATCC 700603 mated at 25°C, Lane 4:* K. pneumoniae* T1, Lane 5:* E. coli* transconjugant of T1 mated at 37°C, and Lane 6:* E. coli* transconjugant of T1 mated at 25°C.

In order to determine the potential transfer of antibiotic resistance genes to other bacteria, conjugal transfer experiments were performed with* K. pneumoniae* T1 and T5 and* S. marcescens* M5 as the donors and sodium azide resistant* E. coli* J53 as the recipient at 25°C and 37°C (Figure 3). The reference strain* K. pneumoniae* ATCC 700603 was used as a positive control. A combination of antibiotic resistance traits was transferred from the donors T1 and T5 to the recipient* E. coli* (CTX CHL GEN CIP from T1 and CTX GEN CIP from T5) when transconjugants were selected on EMB plates containing ciprofloxacin. The frequency of conjugal transfer of pOU11 plasmid was 10^−4^ for both the* K. pneumoniae* donors into the recipient* E. coli* J53 Az^r^. However, the transfer of antibiotic resistance from* S. marcescens* M5 to* E. coli* was not successful.

### 3.5. Southern Hybridization Analysis of Plasmids

**Figure 4:**
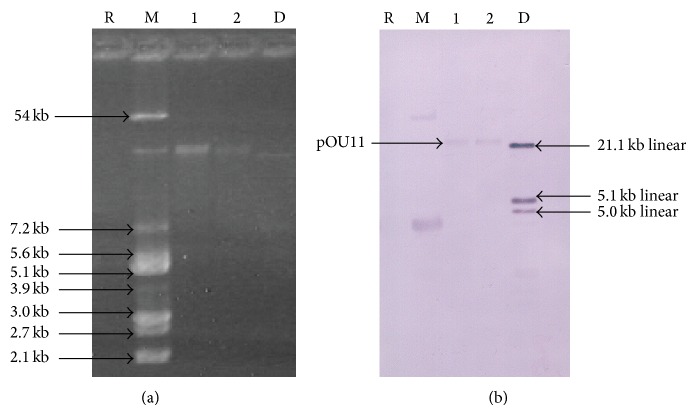
Gel electrophoresis and Southern hybridization of donors and transconjugants. (a) DNA isolated from an environmental strain of* K. pneumoniae* and reference strains of* E. coli* and* K. pneumoniae* and (b) Southern hybridization of an environmental strain of* K. pneumoniae* using the *bla*_SHV_ probe. Lane R: reagent control, Lane M:* E. coli* V517 plasmids, Lane 1:* K. pneumoniae* ATCC 700603 plasmids (used as a reference), Lane 2:* K. pneumoniae* T1 plasmids, and Lane D: DIG-labeled DNA ladder III (Roche), a linear DNA.

The confirmation of the transfer of antibiotic resistance genes on a plasmid was done by plasmid analysis on a 1.0% agarose-TAE gel (Figure 4). Both the donors and the transconjugants carried plasmids of the same size. Similar results were seen for the other ESBL-containing* K. pneumoniae* isolate T5. The presence of a gene encoding an SHV-type ESBL was confirmed to be on a plasmid (pOU11) on the environmental isolate T1 with Southern blotting (Figure 4(b)) and comparison with the reference strain* K. pneumoniae* ATCC 700603.

### 3.6. Pulsed Field Gel Electrophoresis of Environmental and Clinical Strains of* K. pneumoniae*

**Figure 5:**
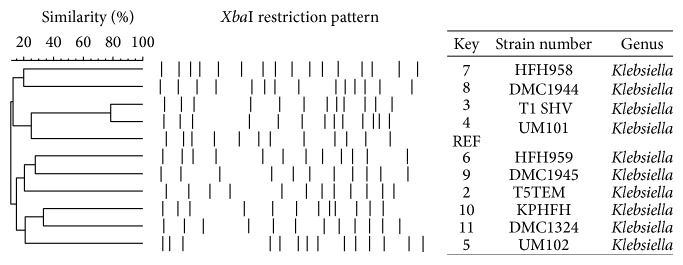
Pulsed field gel electrophoresis of clinical and environmental strains of* K. pneumoniae*. Strain HFH958 is an ESBL-positive clinical strain isolated from a wound; strain DMC1944 is an ESBL-positive clinical strain isolated from urine; strain T1 SHV is one of the ESBL-positive environmental strains discussed in this study; strain UM101 is an ESBL-negative clinical strain isolated from lungs; REF refers to the control strain* K. pneumoniae* ATCC 700603; strain HFH959 is an ESBL-positive clinical strain isolated from sputum; strain DMC1945 is an ESBL-positive clinical strain isolated from sputum; strain T5TEM is one of the ESBL-positive environmental strains discussed in this study; strain KPHFH is an ESBL-positive clinical strain; DMC1324 is an ESBL-negative clinical strain; UM102 is an ESBL-negative strain isolated from urine. All these* K. pneumoniae* strains were isolated in 2011.

Pulsed field gel electrophoresis of the environmental* K. pneumoniae* strains was conducted to determine the clonal relationship between these strains and a variety of clinical strains. As shown in Figure 5, genomic DNA of each isolate was spliced into 11 to 16 fragments and none of them showed similar PFGE pattern in the bands. Therefore, despite the relatedness of the ESBL gene sequences, the environmental strains of* K. pneumoniae* isolated in this study are of different clonal types compared to clinical strains.

## 4. Discussion

Despite the high percentage of ARB on IBL, we found a very low prevalence of ESBL producers which is consistent with previous studies on green leafy vegetables [23, 24, 38]. Similar to our findings that* K. pneumoniae* on IBL harbors ABR genes on self-transmissible plasmids, others have also shown the location of ABR genes on self-transmissible plasmids [23]. Interestingly, we found that* K. pneumoniae* isolate, T1, contained a unique ESBL gene sequence (SHV-type) that was for the first time detected in USA. The detection of this unique SHV-type gene sequence in this pilot study suggests that the IBL is a reservoir of emerging and novel ESBL genes. Moreover, the SHV gene sequence was 99% identical to a SHV-11 gene sequence from a clinical isolate of* K. pneumoniae* in Austria (GenBank accession AEZ49553). This gene sequence is also 99% similar to a gene sequence isolated from* Enterobacter cloacae* from a domestic dog, indicating that these sequences are widely disseminated (Figure 1). For example, Mesa and colleagues detected ESBL-producing bacteria belonging to family Enterobacteriaceae in human feces, wastewater, animal farms, and food [33]. They determined that the occurrence of ESBL-producing enterobacteria in food was relatively low, 0.4% compared to 2.1% in this study. However, it must be noted that the Enterobacteriaceae studied by Mesa et al. were isolated primarily from cooked foods [33].

The first descriptions of ESBL genes involved point mutations of TEM- and SHV-type enzymes produced by clinical isolates, but the prevalence of CTX-M-type enzymes (which preferentially cleave cefotaxime over ceftazidime) has increased dramatically in the last two decades [17]. These three types of ESBL enzymes are considered the most prevalent and the most mutable, as there are now over 170 characterized SHV-type enzymes, 200 TEM-type enzymes, and 130 CTX-M-type enzymes. CTX-M-type enzymes are thought to have originated in an environmental strain of* Kluyvera* spp. and are frequently produced by environmental isolates. For instance, Raphael et al. found that gene sequences in Gram-negative saprophytes on spinach were 100% identical to previously recognized CTX-M-type gene sequences from clinical isolates [38]. Our data also supports these findings, as we showed the presence of CTX-M-1-type beta-lactamase gene sequence in* Serratia marcescens*. Our phylogenetic analysis revealed that the IBL SHV-11 sequences did not match with know SHV genes (Figure 2).

To our knowledge, this is the first report of ESBL-producing enterobacteria on IBL retailed in Michigan markets in USA. Raphael et al. observed an ESBL incidence rate of 2.3% among bacterial isolates from spinach [38] and Bezanson et al. detected ESBL activity in 1.9% of the bacteria isolated from lettuce [22]. In contrast to previous studies on raw salad vegetables where low frequencies of multidrug resistant bacteria were observed (0–23%), we found that the vast majority of our isolates were resistant to multiple antibiotics [22]. These studies excluded species with intrinsic resistance from antibiotic susceptibility tests, such as* Klebsiella* species from ampicillin susceptibility tests [34]. However, in other studies where isolates are not excluded on the basis of intrinsic resistance, high (95%) frequencies of multidrug resistant bacteria were seen on spinach [23]. Despite the high incidence of multidrug resistant bacteria on our IBL samples, we did not observe a high abundance of aminoglycoside or TET resistance in contrast to a recent study conducted in Costa Rica [39]. Rodriguez et al. observed proportional abundance rates of cultivable oxytetracycline-resistant and GEN-resistant isolates between 10% and 100%, but this abundance of antibiotic resistance can be explained by the regular application of GEN and oxytetracycline to the soil on these farms [39]. However, these antibiotics are not used regularly in the United States for the production of vegetables [6] and therefore we observed much lower occurrence of aminoglycoside and tetracycline resistance. Although these antibiotics are not used directly in agriculture, untreated manure or irrigation water containing these antibiotics has been shown to be a possible source for antibiotic resistance in bacteria on green leafy vegetables like iceberg lettuce [24]. In addition to high frequencies of ARB, iceberg lettuce has been implicated in multiple outbreaks of foodborne illnesses in Europe, particularly salmonellosis and shigellosis [12, 40]. Previous outbreaks have involved completely susceptible strains of foodborne pathogens [12]. However, further investigation revealed that several other fecal coliforms present on the source iceberg lettuce were ARB [12]. The presence of these bacteria and their antibiotic resistance determinants on IBL, which is commonly consumed rawly in hospital settings, is the cause for concern, since the ESBL-positive* K. pneumoniae* strains isolated in this study could act as nosocomial pathogens, particularly among immunocompromised patients [35]. The transferability of pOU11 encoding antibiotic resistance at 25°C and 37°C suggests a potential for the resistance to be transferred to other foodborne pathogens such as* Salmonella*,* Shigella*,* Aeromonas*,* Vibrio,* and* E. coli* O157:H7 in hospital and environment settings. Previous studies have shown the thermosensitivity and transferability of antibiotic resistant plasmids at wide range of temperatures are because of formation of temperature dependent synthesis of pili. Since fresh produce (including lettuce) is often served at room temperature (25 degrees Celsius), we studied whether this temperature allows for the transferability of DNA through mechanisms such as increased pili formation or type IV secretion systems [41]. At 37°C the virulence genes are derepressed [42] and our pOU11 plasmid effectively transfers at both temperatures (Figure 3). More research is needed to determine the exact mechanism of evolution and transfer of novel infectious genes expressing virulence properties and antibiotic resistance in ready-to-eat food.

## Figures and Tables

**Table 1:** Primer sequences used in PCR amplification of ESBL and 16S rRNA gene sequences.

Target gene	Primer sequences (5′-3′)	*T*_*m*_ (°C)	Expected amplicon size (bp)	Reference
16S rRNA	F: AGAGTTTGATCMTGGCTCAG	60	1400	[38]
	R: AAGGAGGTGATCCAGCC			

*bla*_SHV_	F: GGTTATGCGTTATATTCGCC	60	867	[33]
	R: TTAGCGTTGCCAGTGCTC			

*bla*_TEM_	F: CCGTGTCGCCCTTATTCC	56	800	[35]
	R: AGGCACCTATCTCAGCGA			

*bla*_CTX-M_	F: TTTGCGATGTGCAGTACCAGTAA	56	500	[35]
	R: CTCCGCTGCCGGTTTTATC			

*bla*_CTX-M-1_	F: AAAAATCACTGCGCCAGTTC	56	415	[35]
	R: AGCTTATTCATCGCCACGTT			

**Table 2:** Identification of 50 representative bacteria from the 138 bacteria selected from iceberg lettuce.

Genus	Number of isolates (%)	Species	Number of isolates (%)
*Pseudomonas *	36 (72%)	*fluorescens *	7 (14%)
		*oryzihabitans *	1 (2%)
		*putida *	5 (10%)
		Unidentified	23 (46%)
*Pantoea *	4 (8%)	*agglomerans *	4 (8%)
*Klebsiella *	2 (4%)	*pneumoniae *	2 (4%)
*Serratia *	2 (4%)	*marcescens *	2 (4%)
*Achromobacter *	2 (4%)	Unidentified	2 (4%)
*Pasteurella *	1 (2%)	*pneumotropica *	1 (2%)
*Suttonella *	1 (2%)	*indologenes *	1 (2%)
*Mannheimia *	1 (2%)	*haemolytica *	1 (2%)
*Cellulomonas *	1 (2%)	*turbata *	1 (2%)

Total	50 (100.00%)		50 (100.00%)

**Table 3:** Multidrug resistance in bacterial isolates from iceberg lettuce.

Number of types antibiotic resistance	Number of isolates (%)	Antibiotic resistance patterns (number of isolates)
0	15 (10.9%)	
1	9 (6.5%)	CTX (3), CIP (2), CHL (2), GEN (2)
2	56 (40.6%)	CTX CHL (52), CTX TET (3), CTX CIP (1)
3	56 (40.6%)	CTX CHL TET (56)
4	2 (1.5%)	CTX CHL GEN CIP (2)
5	0 (0%)	
